# Recurrence of an Oval Window Rupture 25 Years After Traumatic Stapediovestibular Luxation Repair: A Case Report of a Novel Cartilage Sealing Technique

**DOI:** 10.7759/cureus.88281

**Published:** 2025-07-19

**Authors:** Kohei Yamahara, Nobuhiro Hakuba, Ichiro Tateya

**Affiliations:** 1 Department of Otolaryngology - Head and Neck Surgery, School of Medicine, Fujita Health University, Toyoake, JPN; 2 Department of Otolaryngology, Teikyo University School of Medicine, Mizonokuchi Hospital, Kawasaki, JPN

**Keywords:** cartilage, nasal blowing, recurrent oval window rupture, soft tissues, stapediovestibular luxations

## Abstract

Stapediovestibular luxations are often caused by trauma to the external ear canal. Sealing the oval window while leaving the stapes in the vestibule is a safer procedure than stapedectomy, considering the risk of additional inner ear damage, but it risks recurrence. We report a rare case of oval window rupture recurrence 25 years after initial surgery and introduce a new method using cartilage to seal the oval window. A 37-year-old woman experienced a recurrence of oval window rupture after nasal blowing. She had a history of left ear ossicular chain dislocations and oval window rupture caused by an ear pick 25 years earlier, initially treated by sealing the oval window with soft tissues while leaving the depressed stapes in place. In the current surgery, a stapedectomy was performed, the oval window was sealed with cartilage, and an incus columella was placed on the cartilage. Complete resolution of vestibular symptoms and improvement in air conduction thresholds were observed. Postoperatively, no recurrence of oval window rupture was observed. Patients with stapediovestibular luxations face a long-term risk of oval window rupture recurrence if sealed only with soft tissues. Cartilage may offer a promising alternative for oval window sealing after stapedectomy due to its flexibility and durability.

## Introduction

Traumatic stapediovestibular luxations are rare, resulting from direct or indirect force applied to the external ear canal. In this type of ossicular dislocation, internal stapes depression leads to oval window rupture, presenting with cochleovestibular symptoms, including sensorineural hearing loss and vestibular symptoms. As case reports on this condition are limited, no established treatment protocol for stapediovestibular luxations exists yet due to the scarcity of reported cases [1]. Treatment options include stapedectomy or leaving the stapes in the vestibule and simply sealing the oval window. The latter option is safer but carries a risk of recurrent oval window rupture within a few years [2]. Herein, we describe a rare case of recurrent oval window rupture that occurred 25 years after the initial surgery and introduce a novel method for sealing the oval window using cartilage.

## Case presentation

**Figure 1:**
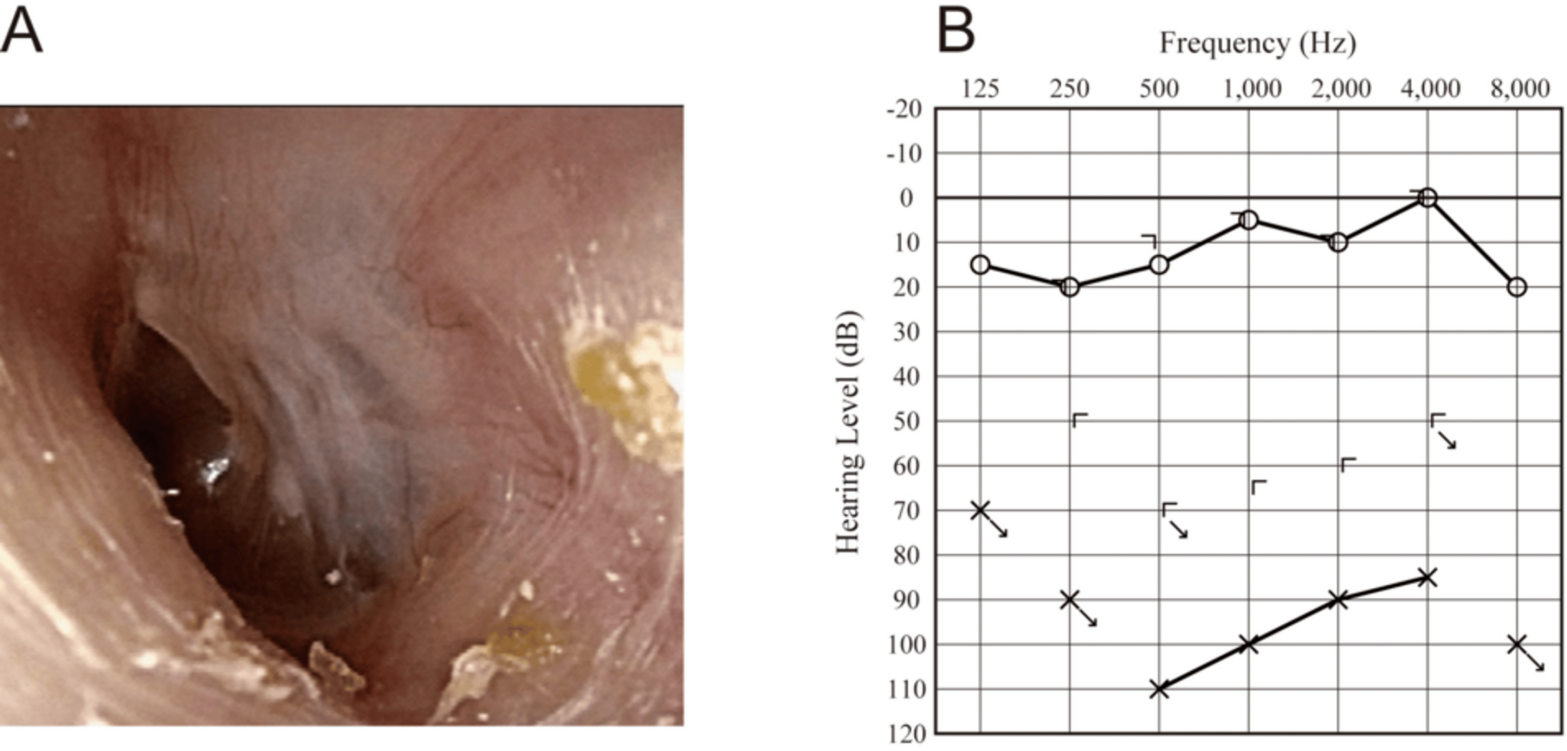
Preoperative otoscopic findings of the tympanic membrane (A) and audiometric evaluation (B).

**Figure 2:**
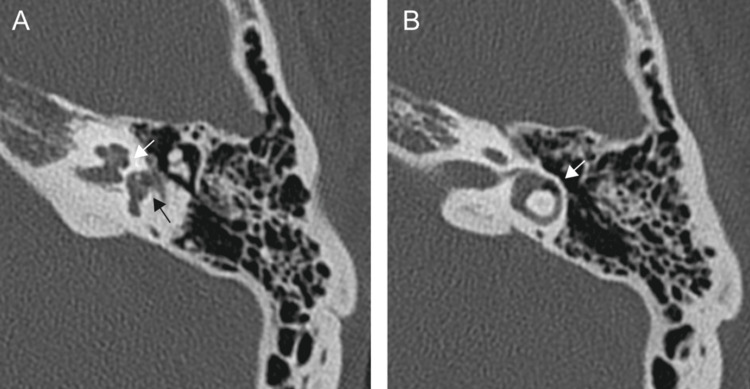
Preoperative CT scans. (A) The black arrow points to the vestibule in which air bubbles and depressed stapes are seen. The white arrow points to the basal turn of the cochlea in which air bubbles are seen. (B) The arrow points to the lateral semicircular canal in which air bubbles are seen.

A 37-year-old woman was transferred to our emergency department with left-sided hearing loss and incapacitating vertigo after nasal blowing. Otoscopic examination revealed an intact tympanic membrane (Figure 1A). Pure-tone audiometry showed severe mixed hearing loss with a pure tone average of 100 decibels (Figure 1B). Computed tomography (CT) scans of the temporal bone revealed a depression of the stapes into the vestibule with air bubbles in the vestibule, lateral semicircular canal, and basal turn of the cochlea (Figures 2A-2B). The patient had a history of left-ear ossicular chain dislocations and oval window rupture from an ear pick injury 25 years earlier. At that time, the patient underwent tympanoplasty at a different hospital, which resolved the vestibular symptoms but not the left hearing impairment. However, as detailed records of the initial surgeries were unavailable, the procedures conducted then remained unclear.

**Figure 3:**
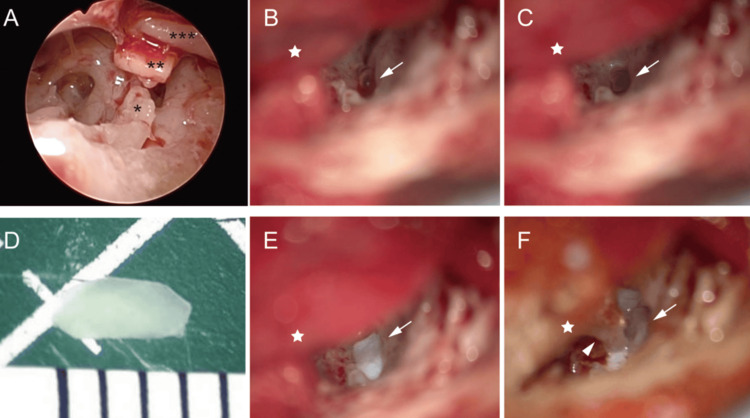
Photographs of the middle ear intraoperatively. (A) Between the depressed stapes (asterisk) and incus (triple asterisks), cartilage (double asterisks) had been inserted at the past surgery, which had been undergone 25 years before the current surgery. (B) Air bubbles in inner ear emerged in the oval window after removing stapes (arrow). (C) Air bubbles were removed by dropping dexamethasone liquid in the oval window. Asterisk points to the oval window. (D) thin-sliced cartilage whose size was 3 mm x 1 mm was prepared. (E) The arrow points to the cartilage placed on the oval window. (F) Incus columella (arrowhead) was placed on the cartilage (arrow). The asterisk indicated elevated tympanic membrane for all figures.

**Figure 4:**
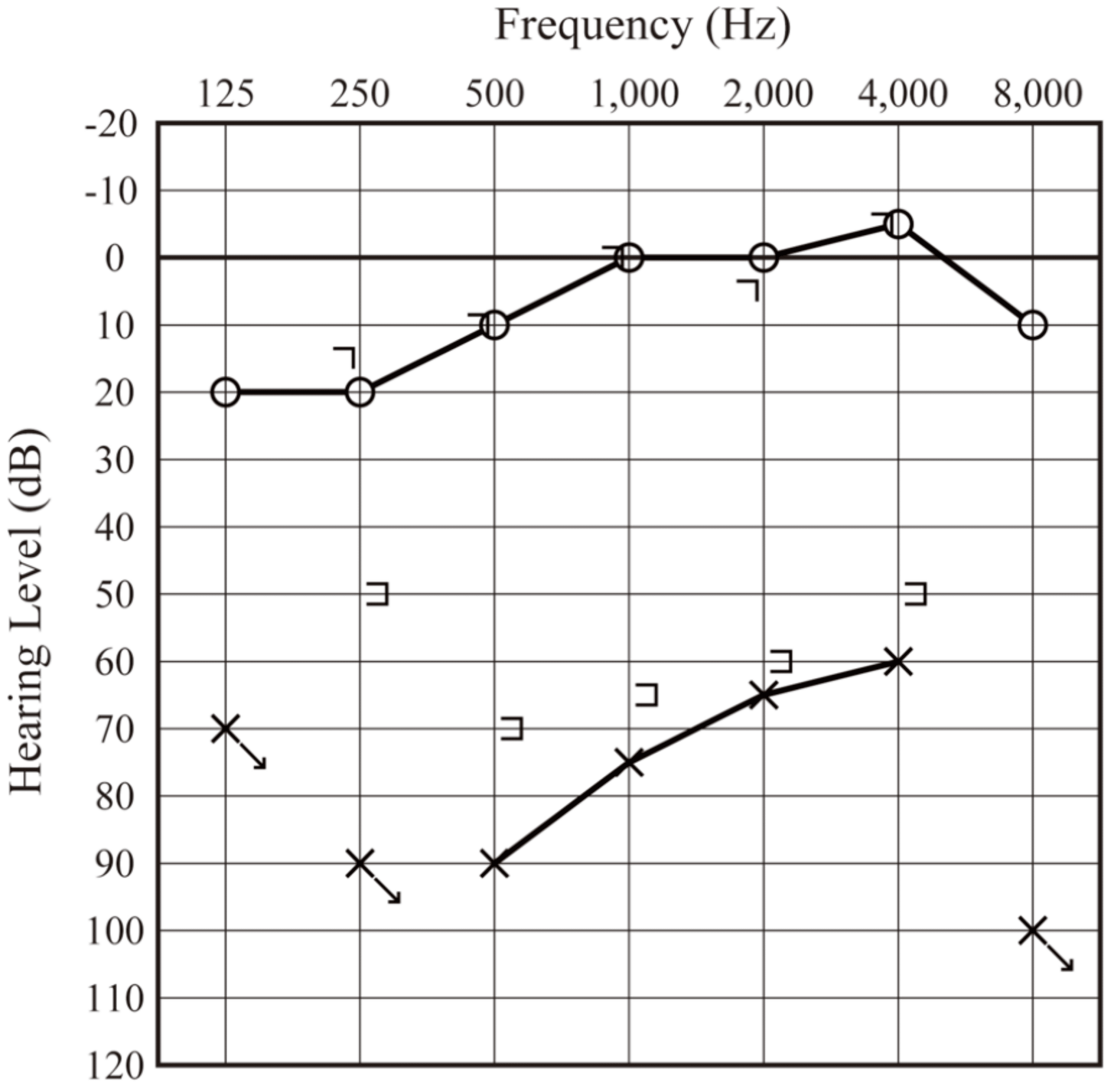
Postoperative audiometric evaluation.

Given the clinical findings, the patient was diagnosed with recurrent oval window rupture. Conservative management, including injection of steroids and antibiotics, bed rest, and avoidance of pressure and straining, was initiated. One week later, the symptoms of vertigo improved, but the hearing level did not, and the air bubbles within the inner ear remained. Given that air bubbles inside the cochlea have been reported to potentially induce inner ear damage, surgical exploration was performed after fully explaining the procedure and its possible complications. After elevating the eardrum, the middle ear cavity was explored, revealing the detailed procedures of the initial surgery. Twenty-five years earlier, earpick trauma had caused dislocation of the incudostapedial joint, depression of the stapes into the vestibule, and rupture of the oval window. During the initial surgery, the oval window was sealed using soft tissues such as connective tissue or fascia, whereas the stapes depression was left unaltered, after which cartilage was inserted between the stapes and incus (Figure 3A). Twenty-five years after the initial surgery, the oval window ruptured again, likely due to the limited durability of the soft material seal, after usual nasal blowing. After confirming the condition in the middle ear, the cartilage between the stapes and incus was removed, and the stapes was gently extracted from the vestibular cavity. The air bubbles in the inner ear emerged in the oval window (Figure 3B) and were removed by applying dexamethasone liquid (Figure 3C). Thin-sliced cartilage with a size of 3 mm × 1 mm was prepared (Figure 3D) and placed on the oval window (Figure 3E), with fat placed around the cartilage. Finally, ossicular reconstruction was performed by placing the incus as a columella on the cartilage (Figure 3F). The dizziness resolved postoperatively. At the five-month follow-up, no additional bone-conductive hearing impairment was observed, and the hearing level improved to pure tone average 76.7 decibels with the closure of the air-bone gap within 10 decibels (Figure 4). CT revealed no residual air bubbles in the inner ear (data not shown). Over two years later, no recurrence of oval window rupture has been observed, even though the patient occasionally blew her nose.

## Discussion

Our report describes a rare case of recurrent oval window rupture 25 years after initial surgery. Moreover, we describe a new method for sealing oval windows using cartilage. The treatment of traumatic internal stapediovestibular luxations is complex. Arragg et al. reported that stapedectomy or repositioning of the stapes is necessary to avoid irreversible inner ear changes [3], whereas Vanderstock et al. recommended avoiding stapes removal or repositioning because removing or lifting up the injured footplate risks causing sensorineural hearing loss [4]. Unfortunately, no established treatment protocol exists for stapediovestibular luxations owing to the limited number of cases [1]. The management of displaced stapes depends on the surgeon’s level of expertise and experience, the condition of the stapes, and the preoperative hearing level. In the initial surgery in our case, leaving the stapes in the vestibule and sealing the oval window are safe and easy procedures. However, this procedure has some limitations. One issue is that leaving the depressed stapes in the vestibule can cause scarring, which may occupy vestibular spaces, potentially leading to late inner ear damage [5]. The recurrence of oval window rupture is relatively common, with rates ranging from 15% [2] to 21% [6]. Recurrence typically occurs within a few months after surgery [2]; however, in our case, recurrence unexpectedly occurred 25 years after the initial surgery. To the best of our knowledge, this report describes the longest interval to recurrence to date. In addition to surgical trauma [7], blast [8], or earpick trauma [9], barotrauma such as forceful sneezing, nasal blowing, or scuba diving can cause oval window rupture through a sudden pressure change in the middle ear [10-12]. In the current case, the recurrence of oval window rupture occurred due to usual nasal blowing, not a forceful event. The patient had a long history of allergic rhinitis and nasal blowing, leading to accumulated damage over the years that might have gradually weakened the oval window. In the current case, the depressed stapes had been in the vestibule for 25 years, and there could be adhesions that connected the depressed stapes and the membranous labyrinth at the time of the current surgery. Removing the stapes would most likely cause additional damage to the membranous labyrinth; thus, sealing the oval window again without lifting the stapes seemed a reasonable option. However, air bubbles remained within the inner ear, and some reports have suggested that air inside the cochlea can potentially induce severe and irreversible hearing loss [13,14]. Therefore, the stapes was removed gently, and subsequently, the air bubbles within the inner ear were removed in the current case, resulting in no additional bone-conductive hearing impairment.

After removing the stapes, the oval window was sealed with a material. Recurrence of oval window rupture after stapedectomy is reportedly rare, although the exact rate is unknown. Surgery is typically not performed after stapedectomy unless symptomatic vertigo suggests a recurrence of oval window rupture. Albera et al. [15] reported a 0.5% incidence rate of delayed vertigo after stapedectomy. Among nine patients who underwent exploration, oval window rupture was identified in three, and all underwent fibrin glue repair, resulting in resolution of vertigo [15]. Different sealing materials have been reported in the literature, mostly utilizing soft tissues such as veins, fat, fascia, perichondrium, and connective tissue [16,17]. A meta-analysis revealed no apparent differences in the postoperative closure of the air-bone gap or the recurrence rate of oval window rupture between various sealing materials [17]. Over the years, many studies have reported cases of traumatic stapediovestibular luxations with stapedectomy and ossiculoplasty with sealing of the oval window using these materials, as summarized in Table 1 [4,9,18-21]. Moreover, improvements in hearing levels were seen in six out of seven cases (Table 1), with no recurrence observed in any case, indicating the usefulness of almost all materials, as highlighted in a meta-analysis [17]. However, no reports have explored the long-term durability of these materials after surgery, suggesting the possibility that the recurrence of oval-window rupture is underestimated [22]. To prevent recurrence in the current case, durable materials that could achieve a tight seal of the oval window were necessary, considering the patient’s habit of nose-blowing. To address these challenges, cartilage was used instead of soft tissues. Thinly sliced cartilage with a size of 1 mm × 3 mm was precisely fitted into the oval window, with small pieces of fat placed around the cartilage to reinforce it. Because of the durability of the oval window sealed with cartilage, no recurrence of oval window rupture has been observed, even though the patient sometimes blew her nose. To the best of our knowledge, this is the first report of the use of cartilage to seal the oval window after stapedectomy. Although cartilage is harder and thicker than soft tissues such as the fascia or connective tissue, it possesses good flexibility, and the sound transduction of cartilage is reportedly efficient [23]. In fact, our case demonstrated postoperative hearing improvements, similar to those in other reports (Table 1). Another advantage of cartilage sealing is that the columella can be placed stably on the oval window instead of using a Teflon wire piston. Yamasoba et al. used the cartilage columella on an oval window sealed with perichondrium, resulting in no improvement in hearing level [18] (Table 1). This failure may be due to the instability of the columella placed on the oval window, sealed with soft tissues. Although long-term observation is required, cartilage could be a possible material for sealing oval windows, considering its flexibility and durability.

**Table 1:** Stapediovestibular dislocation treated with stapedectomy and ossiculoplasty

Author	Cause	Ossicular reconstruction after stapedectomy	Materials for sealing OW	Postoperative hearing	Follow-up periods	Recurrence
Kobayashi et al.[19]	Penetrating trauma with an earpick	malleovestibulopexy	Fascia	Improvement	3 years	No
Chujo et al.[20]	Blow to the opposite side of the head	Teflon piston® on intact incus	-	Improvement	-	No
Vanderstock et al.[4]	Penetrating trauma with a knitting needle	Teflon piston® on intact incus	Vein	Improvement	3 years	No
Yamasoba et al. [18]	Penetrating trauma with an earpick	Cartilage columella between sealed OW and TM	Perichondrium	No improvement	6 months	No
Ishida et al.[21]	Penetrating trauma with the tips of a comb	Connective tissue between the sealed OW and incus	Connective tissue	Improvement	-	No
Ren et al.[9]	Penetrating trauma with an earpick	Reposition of the stapes	Perichondrium	Improvement	5 months	No
Our case	Barotrauma	Cartilage columella between sealed OW and TM	Cartilage	Improvement	2 years	No

## Conclusions

Choosing operative treatment for traumatic internal stapediovestibular luxations is challenging. Patients whose oval windows are simply sealed with materials with the stapes left in the vestibule may have a lifelong risk of oval window rupture recurrence. Stapedectomy should be considered depending on the condition of the stapes, preoperative hearing level, and the surgeon’s skill level and experience. In the present case, sealing the oval window with cartilage resulted in good outcomes in terms of durability and postoperative hearing. Cartilage may be a viable option for sealing oval windows after a stapedectomy; however, further case accumulation and long-term follow-up are needed to validate this approach.
